# Recent Advances on Functionalized Upconversion Nanoparticles for Detection of Small Molecules and Ions in Biosystems

**DOI:** 10.1002/advs.201700609

**Published:** 2018-01-09

**Authors:** Bin Gu, Qichun Zhang

**Affiliations:** ^1^ School of Materials Science and Engineering Nanyang Technological University 50 Nanyang Avenue Singapore 639798 Singapore; ^2^ Division of Chemistry and Biological Chemistry School of Physical and Mathematical Sciences Nanyang Technological University 21 Nanyang Link Singapore 637371 Singapore

**Keywords:** biodetection, energy acceptor, ions, small molecules, upconversion

## Abstract

Significant progress on upconversion‐nanoparticle (UCNP)‐based probes is witnessed in recent years. Compared with traditional fluorescent probes (e.g., organic dyes, metal complexes, or inorganic quantum dots), UCNPs have many advantages such as non‐autofluorescence, high chemical stability, large light‐penetration depth, long lifetime, and less damage to samples. This article focuses on recent achievements in the usage of lanthanide‐doped UCNPs as efficient probes for biodetection since 2014. The mechanisms of upconversion as well as the luminescence resonance energy transfer process is introduced first, followed by a detailed summary on the recent researches of UCNP‐based biodetections including the detection of inorganic ions, gas molecules, reactive oxygen species, and thiols and hydrogen sulfide.

## 1 Introduction

The advancement of biological science is highly depended on new analytical technologies. Especially in the detection of intracellular and intercellular substances, new analytic skills are highly desirable because many molecules in biosystems only display weak signals. One way to address this issue is to introduce some special labels into biosystems. In fact, the strategy to develop new biolabels for the enhancement of biosignals has become one of the hottest areas in recent years.

Among all sensing technologies, photon‐signal‐based biosensors such as organic dyes,^1^, ^2^, ^3^, ^4^ metal complexes,^5^, ^6^, ^7^, ^8^, ^9^, ^10^ and semiconductor nanocrystals^11^ are the most commonly used ones. However, these probes have several limitations in detection or sensing. For example, the emission lifetimes of organic dyes are normally very short (less than 100 ns),^12^ which is very difficult to differentiate the target signal from other short‐lived noises, while quantum dots have been demonstrated to display high toxicity and short circulation half‐time in biosystem.^13^ To overcome these limitations, many research groups found that upconversion nanoparticle (UCNP)‐based probes are promising and could be one of solutions. Compared with conventional fluorescence probes, UCNPs have many charming factors such as high chemical stability,^14^, ^15^ non‐autofluorescence from biosamples,^16^, ^17^, ^18^ large light penetration depth,^19^, ^20^, ^21^ long lifetime (millisecond scale),^22^, ^23^ and less damage to samples.^24^, ^25^, ^26^, ^27^, ^28^

Upconversion refers to a nonlinear optical process, where two or more low energy photons are absorbed through intermediate long‐lived energy states, followed by emitting a high‐energy photon. For traditional fluorescence process, the energy level of excitation photons is higher than that of emission ones. However, in upconversion progress, the energy level of emission photons is higher than that of excitation photons, namely, low‐energy‐excited photons are converted into high‐energy ones. Although this phenomenon has been known more than 60 years,^29^ for a long time, the application of this phenomenon was only limited to bulk glass and crystalline materials.^30^, ^31^ Until the late 1990s, the advance of nanotechnology provided new opportunities to UCNPs and these materials have become one of the most attractive research fields within nanoscience community including many progresses in biodetection.^32^, ^33^, ^34^, ^35^, ^36^

**Figure 1:**
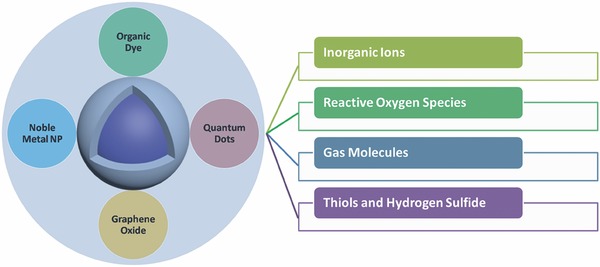
UCNP‐based probes with different energy acceptors to detect different inorganic ions, reactive oxygen species, gas molecules, and thiols.

Most of biodetection studies are based on the luminescence resonance energy transfer (LRET) mechanism. LRET is a radiative process that happens between an energy donor and an energy acceptor. Because UCNPs themselves can only change their emission wavelength through doping and they have no any recognition moieties on their surface, it is unlikely for these nanoparticles to display efficient sensing behaviors. To address this issue, special recognition groups are required to be attached onto the surface of UCNPs, where the optical signals produced by UCNPs will be affected through LRET process. In fact, UCNPs are ideal energy donors, and suitable recognition groups as energy acceptors are required to be attached onto the surfaces of UCNPs to achieve highly efficient and sensitive LRET. Till now, a handful of materials (such as organic dyes, noble metal nanoparticles, graphene oxide, and quantum dots) have been demonstrated as promising energy acceptors (**Figure** **1**).

Because of the fast exploration of UCNPs, several reviews have already summarized the preparation and applications of UCNPs.^23^, ^37^, ^38^, ^39^, ^40^, ^41^, ^42^, ^43^, ^44^, ^45^ In this review, we mainly focus on recent achievements on the usage of lanthanide‐doped UCNPs as efficient probes for biodetection since 2014. We will introduce the mechanisms of upconversion as well as the luminescence resonance energy transfer process first, followed by a detailed summary on the recent researches of UCNP‐based biodetections including the detection of inorganic ions, gas molecules, reactive oxygen species, and thiols and hydrogen sulfide (Figure 1).

## 2 Upconversion and LRET Process

### 2.1 Mechanisms of Upconversion

Most of UCNPs compose of lanthanide ions. The electron configuration of lanthanide ions is 4f*^n^*5s^2^5p^6^ (*n* = 0–14). The partially filled 4f shell is responsible for the multiple energy states in the range of NIR, visible (Vis), and ultraviolet (UV) spectrum. Because the inner 4f electrons are shielded by the outer 5s and 5p electrons, the electronic transitions are almost not affected by the surrounding environment. Thus, energy levels of each lanthanide ion keep constant and these energy levels can produce a group of unique sharp emission peaks like spectroscopic fingerprints.

There are three main upconversion mechanisms: (a) excited‐state absorption (ESA), (b) energy transfer upconversion (ETU), and (c) photon avalanche (PA),^46^, ^47^, ^48^ which are discussed in detail in the following sections.

#### 2.1.1 Excited‐State Absorption

Excited‐state absorption refers to the process that a ground level (G) ion successively absorbs two pump photons and is excited to the E2 level, followed by emitting one photon and returning to ground level again. Two factors ((1) similar energy gap from G to E1 and from E1 to E2, and (2) long lifetime of the intermediate level E1) play key roles in this process. When an ion is excited to the E1 level, it still can accept another photon with the same wavelength to be promoted to the E2 level. To increase the efficiency of ESA, lanthanide ions with ladder‐like energy states are required. Only a few lanthanide ions such as Er^3+^, Ho^3+^, and Tm^3+^ have such energy level distribution.^29^

#### 2.1.2 Energy Transfer Upconversion

Energy transfer upconversion is quite different from ESA because ESA is only operated on one single ion while ETU involves two neighboring ions. In an ETU process, ion 1 as a sensitizer can absorb a pump photon and will be excited to the intermediate level E1. Since level E1 is not very stable, ion 1 can transfer this energy to a neighboring ion 2 (an activator) and ion 1 returns to the ground state. At the same time, ion 2 is excited to E1 level. The energy transfer process can happen again and ion 2 will be excited to its upper emitting state E2, followed by emitting a converted photon. The upconversion efficiency of ETU is strongly related to the average distance between the sensitizer and the neighboring activator, which is determined by the concentrations of these dopants (ion 2). The ETU process is of great importance for UCNPs because most efficient UCNPs to date are based on ion pairs of sensitizers and activators such as Yb^3+^/Tm^3+^, Yb^3+^/Er^3+^, and Yb^3+^/Ho^3+^.

#### 2.1.3 Photon Avalanche

For PA process, the excited energy should be higher than a certain threshold value (E2 − E1). The PA is a looping process, which begins with the promotion of ion's energy from level E1 to level E2 by ESA process. Then, an efficient cross‐relaxation process happens between E2 level ion and another ground state ion. Both ions fill into intermediate E1 state through the following equation: ion 1 (E2) + ion 2 (G) → ion 1 (E1) + ion 2 (E1). The net effect of this loop is that one ion at level E1 produces two ions at level E1, and these two ions are ready to fill into level E2 for further looping process. When the loop ensues, two ions will produce four, and four will produce eight, and so on. Eventually, this progress will lead to the exponential increasing of level E2 ions, like an avalanche. In addition, it is easy to identify PA process because this process usually requires an excitation threshold and a long time (seconds) to build up.

### 2.2 LRET‐Based Detection Strategy

LRET is a powerful spectral technique to study interactions between nanoparticles. This unique property of UCNPs has greatly promoted the development of their applications in biodetection. The core of upconversion detection is based on different LRET efficiency of a probe before and after reacting with target analytes. To trigger the LRET process in detection, the energy acceptor should meet two requirements: (1) the absorption band of the acceptor should overlap with the emission band of UCNPs; and (2) the acceptor should be close enough to UCNPs. There are two major approaches (tuning the spectral overlap (1) and distance (2) between UCNPs and the acceptors) to manipulate LRET efficiency.

#### 2.2.1 Tuning Spectral Overlap

**Scheme 1:**
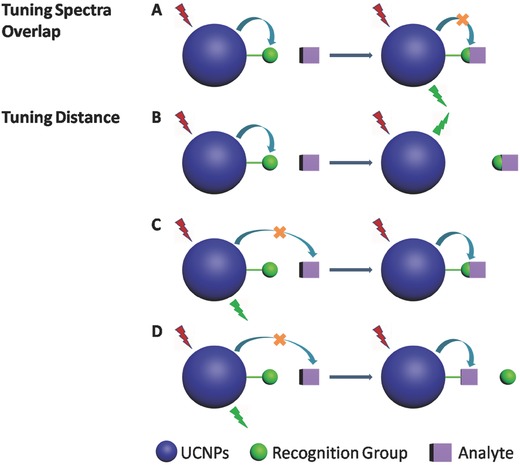
Main LRET‐based detection strategies

To manipulate the LRET efficiency, the absorption of acceptors should show a significant change (either intensity or wavelength) after reacting with analyte. As shown in **Scheme** **1**A, the analyte will react with recognition groups to block the LRET process, leading to the recovery of the emission of UCNPs. It should be noticed that this detection strategy is also effective on the opposite direction; in other words, both triggering and blocking the LRET process are effective. Because a variety of fluorescence probes^49^ have been reported to detect ions and small molecules through the changes in absorption or fluorescence, it is a very convenient way to construct an LRET‐based probe through attaching suitable sensing groups onto the surface of UCNPs.

#### 2.2.2 Tuning Distance

The other strategy is to change the distance (lengthening or shortening) between UCNPs and the acceptors. In Scheme 1B, the recognition group acts as an energy acceptor. The addition of analytes will cut the linkage between UCNPs and acceptors, leading to the recovery of the emission. However, in Scheme 1C,D, the analytes act as energy acceptors, which will either combine with (in Scheme 1C) or replace (in Scheme 1D) the recognition groups. These strategies are also effective on both directions.

The LRET process could be improved based on these approaches. Scientists employ quantum dots (QDs) as energy acceptors and the emission of UCNPs as the excitation sources for QDs. This additional QD emission signal provides various potential applications of this probe during detections.

### 2.3 Biotoxicity of UCNPs

It becomes more and more important to assess their potential hazards when UCNPs have been widely used in biosystem. Generally, the bioapplications of UCNPs can be divided into two parts: in vitro and in vivo. In vitro test refers to the test conducted in living cells such as Hela cells and KB cell, while in vivo refers to the test conducted in living animals such as rats and zebra fish.

To evaluate the cytotoxicity of UCNPs, one of the most common tests is MTT assay, which is rapid, standardized, sensitive, and low‐cost. To conduct this test, UCNPs are first incubated into living cells with different conditions, and then the viability of the cells is recorded to judge the biotoxicity of the UCNPs.

Several research results have already shown that UCNPs almost have no cytotoxicity to various cell lines. For example, Li and co‐workers reported that Yb/Er‐doped UCNPs with silica shell did not show obvious effect on KB cells after the incubation with a concentration of 800 µg mL^−1^ UCNPs for 20 h.^50^ Shan et al. demonstrated that COOH or NH_2_‐functionalized UCNPs display a limited toxicity to human osteosarcoma cells after incubation for 9 d.^51^ In vivo tests of cytotoxicity were also conducted through animal studies. For example, Jalil and Zhang reported that healthy rats with the injection of silica‐coated UCNPs (10 mg kg^−1^) did not display any abnormal behavior after 7 d.^52^ In addition, they also investigated the distribution of UCNPs in different organs and found that most of the UCNPs were excreted through urine after 7 d.

## 3 UCNP‐Based Nanoprobes for Different Analyte Detection

In this section, we discuss UCNP‐based probes since 2014 according to their different applications including the recognition of inorganic ions, the sensing of reactive oxygen species, the identification of gas molecules, and the detection of thiols and hydrogen sulfide.

### 3.1 The Recognition of Inorganic Ions

Although the concentrations of inorganic ions in biosystems are not very high, they do play key roles in metabolism. Thus, it is highly desirable to efficiently monitor them. In this section, we discuss them in detail according to individual ions.

#### 3.1.1 Ag^+^ Sensors

**Figure 2:**
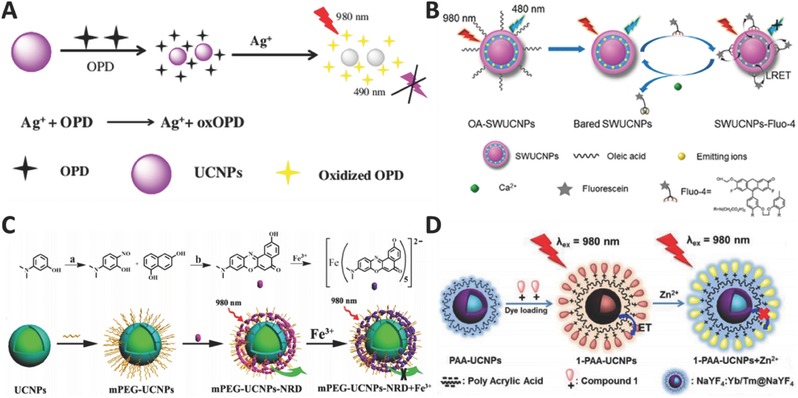
Schematic illustration for detection of A) Ag^+^ (Reproduced with permission.^53^ Copyright 2017, Springer‐Verlag), B) Ca^2+^ (Reproduced with permission.^55^ Copyright 2015, American Chemical Society), C) Fe^3+^ (Reproduced with permission.^56^ Copyright 2016, American Chemical Society), and D) Zn^2+^ (Reproduced with permission.^57^ Copyright 2015, American Chemical Society).

Due to its wide application in photography imaging industry and electrical industry, thousands of tons of Ag^+^ as industrial wastes are released into the environment each year. Clearly, such amount has already caused serious environmental problems. Therefore, it is of great importance to monitor Ag^+^. Recently, Yao and co‐workers developed a new UCNP‐based probe to address this problem.^53^ When they mixed Ag^+^, UCNP probe, and *o*‐phenylenediamine (OPD) in aqueous solution, they found that Ag^+^ can oxidize OPD to form oxOPD, which can efficiently quench the emission of UCNPs (**Figure** **2**A). The Ag^+^ ions can be quantitatively detected within the range between 0 and 0.5 × 10^−3^ m with the detection limit of 33 × 10^−9^ m. Zhu and co‐workers fabricated another Ag^+^ detection platform with the detection limit of 60 pm,^54^ where they functionalized UCNPs with amino‐labeled single‐stranded DNA to capture Ag^+^, and employed graphene quantum dots (GQDs) as energy acceptors. The original upconversion luminescence (UCL) of the as‐prepared probe is quenched due to the strong interaction between DNA and GQD; however, upon the addition of Ag^+^, this reaction can become weak leading to the recovery of UCL.

#### 3.1.2 Ca^2+^ Sensors

Ca^2+^ is an important inorganic ion that human beings require to prevent osteoporosis, and to maintain healthy blood pressure levels, a healthy nervous system, and strong bones and teeth. Its deficiency in body could cause many diseases. Thus, how to efficiently and selectively monitor its level in biosystem is highly desirable and urgent. Liu and co‐workers constructed a high efficient upconversion nanoprobe to detect and bioimage Ca^2+^.^55^ This probe has a sandwich structure with a core‐inner shell‐outer shell architecture decorating energy acceptors on its surface (Figure 2B). The emitting ions are located at the inner shell, and the oleic acids on the surface are removed to further shorten the distance between energy donors and acceptors. Upon the addition of Ca^2+^, the emission quenched by the acceptor will gradually recover and the detection limit can reach 15 × 10^−12^ m. It should be pointed that this strategy can become a general method to sense other analytes by simply changing the acceptors.

#### 3.1.3 Fe^3+^ Sensors

Fe^3+^ is the most abundant trace metal ion in human body and is closely related to many physiological processes. Shi and co‐workers reported a novel Gd^3+^‐doped UCNPs probe for the detection of Fe^3+^ in living cells.^56^ They attached a novel Nile red derivative onto the surface of PEG‐modified UCNPs. Upon the addition of Fe^3+^, the emission of UCNPs is quenched by the dye–Fe^3+^ complex (Figure 2C). Moreover, the Gd^3+^ in UCNPs endows the probe with an effective T_1_ signal enhancement, making it a promising magnetic resonance imaging (MRI) contrast agent.

#### 3.1.4 Zn^2+^ Sensors

Developing an efficient detection system to recognize Zn^2+^ in organisms is highly important due to its crucial role in metabolism. Chang and co‐workers reported a rational design to detect Zn^2+^.^57^ They found that UCL could be effectively quenched by the dyes on the surface of UCNPs and can be subsequently recovered by the addition of Zn^2+^ (Figure 2D). Moreover, this nanosystem can be applied in mouse brain slice with Alzheimer' s disease and zebra fish. In another research group, Zhang and co‐workers demonstrated a multifunctional UCNPs probe not only for in situ detection of endogenous Zn^2+^, but also to control ^1^O_2_ release guided by Zn^2+^ with highly therapeutic performance in living cells.^58^ Their results provide a new insight to develop and precisely control photodynamic therapy (PDT) through the detection of Zn^2+^, and promote PDT to be safer and more attractive clinical technique for cancer treatment.

#### 3.1.5 Cu^2+^ Sensors

**Figure 3:**
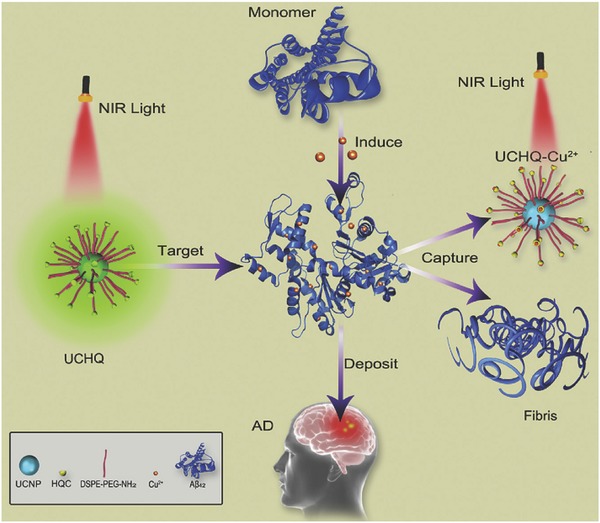
Schematic illustration for detection of Cu^2+^ in mice model with Alzheimer's disease. Reproduced with permission.^61^ Copyright 2016, Elsevier B.V.

Cu^2+^ is the third abundant trace metal ion in human body and many researches on Cu^2+^ detection have been reported. Cai and co‐workers constructed a novel probe by assembling dyes onto SiO_2_‐coated upconversion nanorods^59^ and found that Cu^2+^ can combine with the dye and quench the UCL. Shi and co‐workers employed the similar strategy to detect Cu^2+^ and applied this detection in living cells.^60^ Almost at the same time, Bu and co‐workers applied the same strategy in mice with Alzheimer's disease^61^ and found that the aggregation of β‐amyloid (Aβ) proteins is highly related to Alzheimer's disease, and more Cu^2+^ ions may strongly promote the aggregation. In addition, they also found that the chelator on the surface of UCNPs can capture Cu^2+^ from the complex (**Figure** **3**). Therefore, this probe can be used not only for the detection and imaging of Cu^2+^ and Aβ, but also for the inhibition of Cu^2+^‐induced aggregation. Xian and co‐workers developed a more complicated system for the sequential detection of Cu^2+^, pyrophosphate (PPi), and alkaline phosphatase (ALP).^62^ The emission will be quenched due to the coordination between Cu^2+^ and polyethyleneimine on the surface of UCNPs. If a more powerful chelator PPi is added, Cu^2+^ will be released from UCNPs, leading to the turn‐on emission. Note that the as‐resulted emission can be turned off again through the addition of ALP.

#### 3.1.6 Cr^3+^ Sensors

Jiang and co‐workers reported a Ce^3+^‐doped UCNP probe for the detection of Cr^3+^.^63^ They control the emission intensity ratio of red/green and manipulate the output color of UCNPs from green to red through changing the concentration of Ce^3+^. Cr^3+^ can selectively bind with dye molecules on the surface of UCNPs and quench their green emission band. The limit of detection was calculated to be 4.1 × 10^−6^ m.

#### 3.1.7 Hg^2+^ Sensors

**Figure 4:**
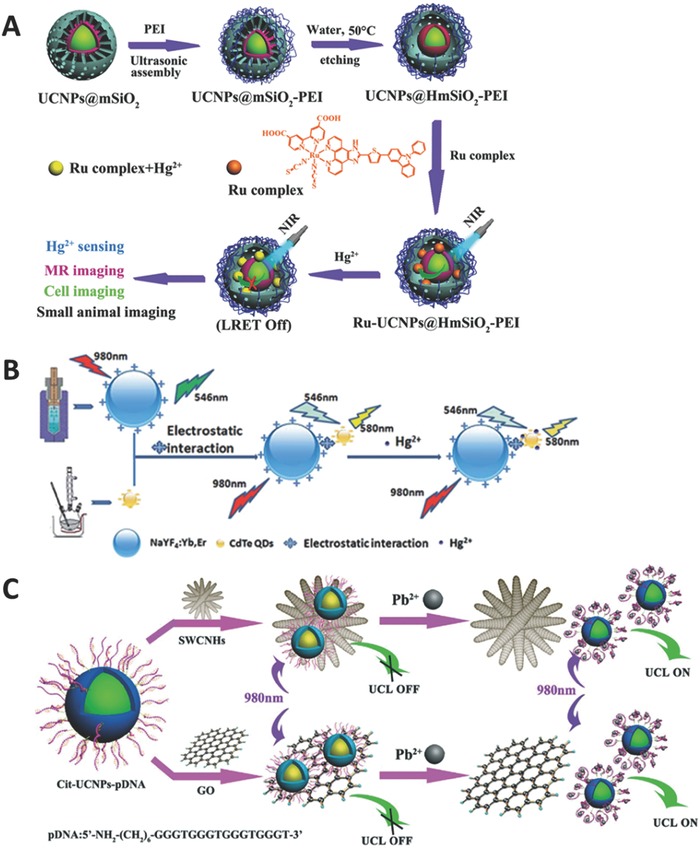
A) Schematic illustration of multichannel detection of Hg^2+^. Reproduced with permission.^66^ Copyright 2015, Royal Society of Chemistry. B) Schematic illustration for Hg^2+^ detection using quantum dots as energy acceptor. Reproduced with permission.^67^ Copyright 2015, Royal Society of Chemistry. C) Schematic illustration for Pb^2+^ detection using single‐walled carbon nanohorns and graphene oxide as energy acceptors. Reproduced with permission.^77^ Copyright 2016, Royal Society of Chemistry.

Hg^2+^ is one of the extremely toxic ions, which can accumulate in human bodies leading to a severe nervous system damage. Therefore, sensitively and efficiently monitoring Hg^2+^ is highly desirable. Feng and co‐workers reported a UCNP probe for the detection of Hg^2+^ in water.^64^ They found that the ruthenium complex on the surface of UCNPs can quench the emission; however, the addition of Hg^2+^ will turn on the emission again since Hg^2+^ can react with the ruthenium complex. The detection limit through the usage of UCL signal is 8.2 ppb, which is much lower than that obtained from the absorption technique. Zhang and co‐workers improved this strategy based on thiazole derivatives as energy acceptors and applied this probe in living cells.^65^ Sun and co‐workers demonstrated a hollow mesoporous silica‐coated UCNPs probe,^66^ which could be acted as an energy acceptor after loading ruthenium complex onto it (**Figure** **4**A). This as‐fabricated probe after Gd^3+^ doping could be further employed to perform MRI in the Kunming mice model.

The energy acceptors used for this detection can be organic dyes, metal complexes, or other nanoparticles. Han and co‐workers employed CdTe quantum dots as energy acceptor,^67^ and found that the QDs can be excited by the UCNP emission (546 nm) and emit 580 nm photons (Figure 4B). The addition of Hg^2+^ will affect the emission intensity at 580 nm with a linear range from 0.01 to 2.8 × 10^−6^ m. Based on the similar strategy, Chen and co‐workers designed and fabricated an upconversion film through self‐assembly during solvent evaporation.^68^ Besides these studies, there are also some other strategies reported in literature. For example, Chu and co‐workers reported a DNA‐functionalized UCNPs probe to sense Hg^2+^ with a detection limit of 5 × 10^−9^ m 69 while some other groups employed rhodamine‐derivative‐functionalized upconversion nanorods to recognize Hg^2+^.^70^, ^71^, ^72^

#### 3.1.8 Cd^2+^ Sensors

Cd^2+^ is another extremely toxic ion, which has already been reported to cause Itai–itai disease in Japan. Thus, it is urgent to develop an efficient probe to monitor it. Recently, Zhang and co‐workers developed a novel UCNP probe to sense Cd^2+^.^73^ In their research, they employed gold nanoparticles (AuNPs) as energy acceptors. To prevent the aggregation of AuNPs, GSH was added to enlarge the interparticle distance between AuNPs and UCNPs, and the as‐prepared probe did not show strong emission. Upon the addition of Cd^2+^, GSH can react with Cd^2+^ to form (GSH)_4_Cd complex, resulting in the aggregation of AuNPs. The as‐aggregated AuNPs are released from the surface of UCNPs and the emission of UCNPs is gradually recovered.

#### 3.1.9 Pb^2+^ Sensors

As the commonly used chemicals for several decades, lead and its products have caused significant environment pollution. Since lead can accumulate in soil and water, the detection of lead is very important. Song and co‐workers reported a UCNP probe with CdTe quantum dots as energy acceptor and applied this probe in human serum for the first time.^74^ They found that QDs can be excited by the emission of UCNPs; however, the addition of Pb^2+^ can gradually quench the emission of QDs. Lv and co‐workers demonstrated a UCNP probe with gold nanoparticles as energy acceptors.^75^ The AuNPs are attached on the surface of UCNPs through electrostatic interactions. Upon the addition of Pb^2+^, the AuNPs will leave the surface of UCNPs and the energy transfer from UCNPs to AuNPs is blocked, leading to the recovering emission of UCNPs. Wang and co‐workers presented a dual detection system to simultaneously detect Pb^2+^ and Hg^2+^.^76^ In their research, they employed AuNPs as energy acceptors, and two kinds of UCNPs with different emission colors as energy donors. The two donor–acceptor pairs were fabricated for different analytes through a special sequence of DNA. In the presence of Pb^2+^ and Hg^2+^, DNAs will bind to their corresponding analytes and formed a stable G‐quadruplex structure for Pb^2+^ or a hairpin‐like structure for Hg^2+^, leading to the separation between UCNPs and AuNPs. Therefore, the dual energy transfer is disrupted and two emission bands (green and red) are recovered. Sun and co‐workers reported another UCNP probe based on the similar strategy.^77^ In their research, they employed DNAs to link UCNPs and acceptors, where the emission can be restored through the usage of G‐quadruplex–Pb^2+^ structure. The difference is that Sun and co‐workers used single‐walled carbon nanohorns or graphene oxides (GO) as energy acceptors (Figure 4C).

#### 3.1.10 CN^−^ Sensors

Substances containing CN^−^ are widely used in industrial processes such as metallurgy and electroplating. However, CN^−^ is very dangerous to human body due to its high affinity to Fe^3+^, and the detection of CN^−^ has attracted considerable attention. Zhu and co‐workers presented a phenothiazine–cyanine‐modified UCNP probe to detect CN^−^ in water samples.^78^ The dye on the surface of UCNPs can quench the emission; however, the addition of CN^−^ will react with the dye, resulting in the recovery of the emission.

#### 3.1.11 F^−^ Sensors

Although F^−^ is one of the most common anions, its excessive intake will lead to serious health problems. Chen and co‐workers reported a curcumin‐derivative‐modified UCNP probe to detect F^−^ in real samples such as tap water and milk.^79^ The surface‐attached dye can react with F^−^ and quench the emission of UCNPs. The detect limit is as low as 5 × 10^−6^ m.

#### 3.1.12 NO_2_^−^ Sensors

The environment is easy to be contaminated by NO_2_^−^ because it can easily be produced through natural or biological conversions of nitrogen‐containing organics such as livestock manure, chemical fertilizers, and natural deposits.^80^ A number of medical issues such as esophageal cancers and blue baby syndrome are related to the chronic intake of nitrite. Therefore, the determination of the NO_2_^−^ level in drinking water and food qualities is crucial. Zhang and co‐workers reported a UCNP‐based probe to detect NO_2_^−^ by using NO_2_^−^‐sensitive dye (neutral red (NR)) as an energy acceptor. During the detection, NO_2_^−^ could react with NR to form diazonium salt, which is not stable and is easy to decompose. The absorption of the as‐resulted product has a blueshifted absorption compared to that of NR, leading to the recovery of green emission band of UCNPs.^81^

#### 3.1.13 pH Sensors

Since Wolfbeis and co‐workers presented first pH probe based on upconversion nanorods,^82^ the advance on optical pH sensor is significant. Zhang and co‐workers reported dye‐modified upconversion nanotubes to detect pH in aqueous solution and they found that the intensity of red emission band is linear to the change of pH value from 6 to 8.^83^ The same group also introduced Eu^3+^ into UCNPs and found that the as‐fabricated probes show a high efficiency to sense pH, which can be applied in physiological pH range.^84^

Schaferling and co‐workers reported another pH‐dependent dye‐modified probe for the application in bioimaging of living cells.^85^ The detection range is between 3.0 and 6.7 with a resolution of 0.3 pH. Then, they further coated the UCNPs with polyethylenimine to improve the cellular uptake properties and found that the as‐prepared probe can distinguish the changes in membrane trafficking.^86^ Later on, the same group also demonstrated another pH‐sensor film, which consists of upconversion particles and pH‐responsive dye.^87^ The highlight of this research is that the pH distribution could be read out by a color camera, which is much cheaper and straightforward. Such probes can be used to measure pH in human serum samples. Besides these, UCNP‐based pH sensor films can also be used to monitor the growth of bacteria.^88^ Additionally, the pH sensor film could be doped with other energy acceptors. For example, Zhao and co‐workers could fabricate a film with graphene oxide as an acceptor,^89^ and they found that the higher the pH, the stronger the interaction between UCNPs and GO is. Recently, dye‐modified UCNPs can also be used as pH probes for the application in living cells. For example, different energy acceptors such as xylenol orange^90^ and fluorescein isothiocyanate^91^, ^92^ have been reported.

### 3.2 Gas Molecules

**Figure 5:**
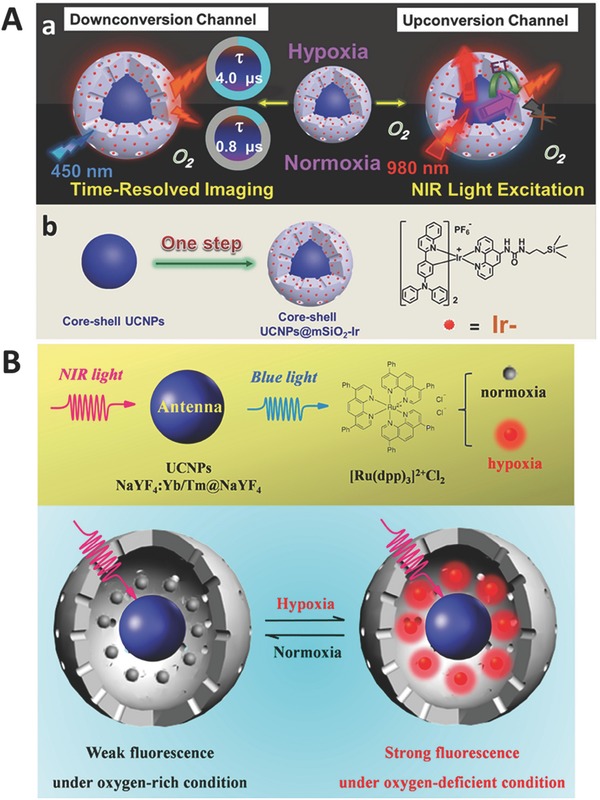
Schematic illustration for O_2_ detection using A) Ir complex (Reproduced with permission.^94^ Copyright 2015, Wiley‐VCH Verlag GmbH & Co. KGaA) and B) Ru complex (Reproduced with permission.^95^ Copyright 2014, American Chemical Society) as energy acceptors.

The research on the detection of gas molecules such as O_2_, CO_2_, and NH_3_ is proceeding very fast because such detections play significant roles in bioanalytical chemistry, clinical medical diagnosis, and bioprocess monitoring.^40^ Wolfbeis and co‐workers first reported UCNP‐based probe with iridium complexes as O_2_ indicators to detect O_2_.^93^ Zhao and co‐workers developed a phosphorescent iridium‐complex‐modified nanoprobe, which could monitor the concentration of O_2_ under both downconversion and upconversion channels (**Figure** **5**A).^94^ In this system, they also imported time‐resolved imaging technique, which is meaningful to detect O_2_ in tissues with nonuniform O_2_ distribution. Shi and co‐workers performed a comprehensive study of a UCNP‐based probe for the detection of oxygen levels reversibly both in vitro and in vivo (Figure 5B).^95^ In their system, ruthenium complexes were employed as oxygen indicators, which can selectively react with oxygen. Lannutti and co‐workers demonstrated an O_2_ probe with a core–shell fiber structure and same ruthenium complexes as energy acceptors.^96^ Song and co‐workers employed platinum complexes as energy acceptors to construct O_2_ probe. The outside of this probe is coated with an ultrathin layer silane,^97^ which affords a hydrophobic environment to hold hydrophobic molecules for the realization of multifunctions.

As one of the signaling molecules, NO plays an important role in several important biological processes. Li and co‐workers reported a UCNP‐based probe to detect NO in biological fluids, living cells, and tissues.^98^ Their probe consists of UCNP core, mSiO_2_ shell, and a rhodamine B derivative. The addition of NO would change the absorption of the chromophore and quench the emission of UCNPs.

### 3.3 Reactive Oxygen Species

Reactive oxygen species (ROSs) such as H_2_O_2_, HClO, and •OH are formed as natural byproducts of normal metabolism and have important effects in cell signaling and homeostasis. However, UV exposure or heat exposure can dramatically increase the levels of ROSs, leading to the remarkable damage to cells including DNA or RNA damage, lipid peroxidation, and amino acids oxidation. Therefore, the detection of the level of ROSs in biosystem is extremely important.^99^, ^100^ Qu and co‐workers reported a novel UCNP‐based probe with dye‐labeled hyaluronic acid (HA) as energy acceptors for the detection of ROSs and bioimaging.^101^ ROSs can induce the degradation of HA chain and subsequently detach HA fragments from UCNPs, which can enable ratiometric UCL as a detection signal. Such a probe has been successfully applied to evaluate early treatment of arthritic mice with antiarthritis drug MTX.

#### 3.3.1 H_2_O_2_ Sensors

**Figure 6:**
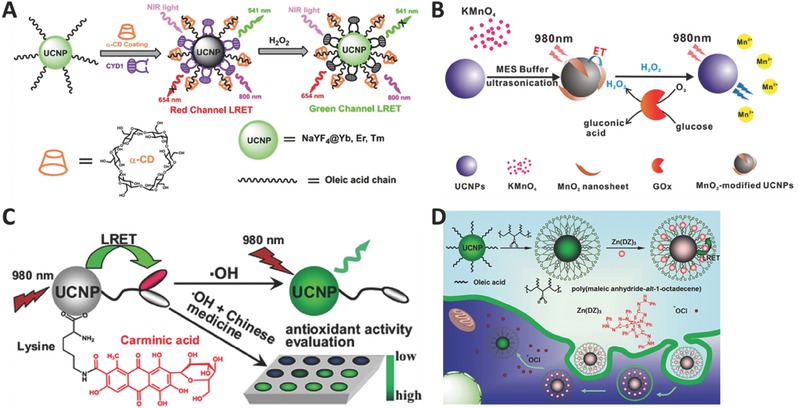
Schematic illustration for H_2_O_2_ detection using A) organic dye (Reproduced with permission.^102^ Copyright 2015, Elsevier B.V.) and B) MnO_2_ nanosheet (Reproduced with permission.^103^ Copyright 2015, American Chemical Society) as energy acceptors. Schematic illustration for detection of C) •OH (Reproduced with permission.^110^ Copyright 2015, Elsevier B.V.), and D) HClO (Reproduced with permission.^113^ Copyright 2015, Wiley‐VCH Verlag GmbH & Co. KGaA).

Endogenous H_2_O_2_ is an important parameter related to cellular signal transduction and homeostasis, while abnormal H_2_O_2_ level in living systems will disturb normal physiological processes. Zhang and co‐workers presented a novel design based on organic dye‐attached UCNPs for the selective detection of H_2_O_2_.^102^ With the addition of H_2_O_2_, red emission band is recovered while green emission band is quenched (**Figure** **6**A). This nanoprobe can be also applied in ratiometric UCL imaging of H_2_O_2_ in living cells and whole‐body animals.

Chu and co‐workers reported another MnO_2_‐nanosheet‐modified UCNP probe for rapid and sensitive detection of H_2_O_2_ and glucose in human serum and whole blood.^103^ In their strategy, MnO_2_ nanosheets are employed to act as energy acceptors (Figure 6B). Because glucose can be oxidized to form H_2_O_2_ through glucose oxidase, this probe is also suitable for the detection of glucose. It should be noticed that this nanoprobe can easily be explored to detect various H_2_O_2_‐involved analytes. Later, the same group developed another H_2_O_2_/glucose probe based on the same conversion from glucose to H_2_O_2_, using DNA‐templated Ag nanoparticles (DNA‐AgNPs) as energy acceptors.^104^ With the addition of H_2_O_2_, AgNPs will be oxidized into Ag^+^ and the original emission is recovered. Other groups also demonstrated an H_2_O_2_/glucose probe based on UCNPs through using organic dye TMB^105^ or squaric acid–Iron complex^106^ as energy acceptors.

#### 3.3.2 •OH Sensors

Hydroxyl radical (•OH) is one of the most reactive ROSs and shows a strong ability to damage biomolecules including nucleic acids, proteins, lipids, and carbohydrates.^107^, ^108^ The detection of •OH in living systems is crucial to understand its physiological and pathological roles. However, the detection is very challenging due to its extremely low concentration and high reactivity in the body. Liu and co‐workers demonstrated a UCNP‐based nanoprobe to detect •OH in vivo for the first time.^109^ They employed azo dyes as energy acceptors to construct a sandwich probe. The as‐fabricated probe shows a higher quench efficiency and the detection limit is 1.2 F m, which is several orders of magnitude lower than most of previous fluorescence probes for •OH detection. Li and co‐workers first reported a simple and effective UCNP probe for the selective detection of •OH and the visual evaluation of •OH‐scavenging activities of drugs.^110^ The working mechanism is as follows: the emission of UCNPs is first quenched by carminic acid (CA), and then it can be recovered through oxidative cleavage of CA in the presence of •OH (Figure 6C). Zhou and co‐workers presented an ultrahigh sensitive multifunctional nanoprobe to detect •OH concentration and further evaluated heavy metal‐induced oxidative stress.^111^ This method has a broad linear detection range from 16 × 10^−12^ m to 2 × 10^−6^ m, and a low detection limit up to 4 × 10^−12^ m.

#### 3.3.3 HClO Sensors

Among all ROSs, HClO is one of the most powerful natural oxidants. HClO is mainly generated through myeloperoxidase enzyme‐mediated peroxidation of chloride ions and plays a key role in response to inflammatory stimuli. However, abnormal level of HClO will damage host tissues and cause many inflammation‐related diseases. Therefore, developing highly sensitive and selective probes to image HClO in living system is highly desirable. Zhang and co‐workers reported a novel UCNP‐based probe through employing rhodamine derivatives as energy acceptors.^112^ More importantly, this probe could be used in ratiometric visualization of HClO release in living cells. Li and co‐workers demonstrated a UCNP probe based on Zn(DZ)_3_ complexes as energy acceptors.^113^ With the addition of HClO, Zn—S—C bonds in Zn(DZ)_3_ complexes will be selectively oxidized, and the emission quenched by the Zn(DZ)_3_ complexes will be recovered (Figure 6D). Furthermore, this probe has been successfully applied to image different amounts of exogenous hypochlorite in living HeLa cells. Feng and co‐workers designed and synthesized a novel Nd^3+^‐doped UCNPs probe using cyanine dyes as energy acceptors.^114^ This as‐fabricated nanoprobe has been applied in the detection of ClO^−^ in a mouse model under 808 nm irradiation. Importantly, using an excitation laser with the wavelength at 808 nm can efficiently reduce the heating effect, comparing with the commonly used 980 nm laser.

### 3.4 Thiols and Hydrogen Sulfide

Thiols are organosulfur compounds that contain an R—SH bond, which is the sulfur analog of alcohols. This part only focuses on the detection of three main biothiols: cysteine (Cys), homocysteine (Hcy), and glutathione (GSH). Cys is an essential proteinogenic amino acid. The thiol group in Cys often acts as nucleophiles in enzymatic reactions, and is easy to be oxidized to form a disulfide derivative, which can serve as an important structural role in many proteins. Due to its high reactivity, thiol group in Cys has numerous biological functions. Hcy is a homologue of Cys, differing by an additional —CH_2_— group. Compared with Cys, Hcy is easier to form a five‐member ring through the cyclization. Because of the self‐looping reaction, Hcy‐containing peptide tends to degrade.^115^ GSH is an important antioxidant in living systems and is capable of preventing the damage to cellular components caused by ROSs.

**Figure 7:**
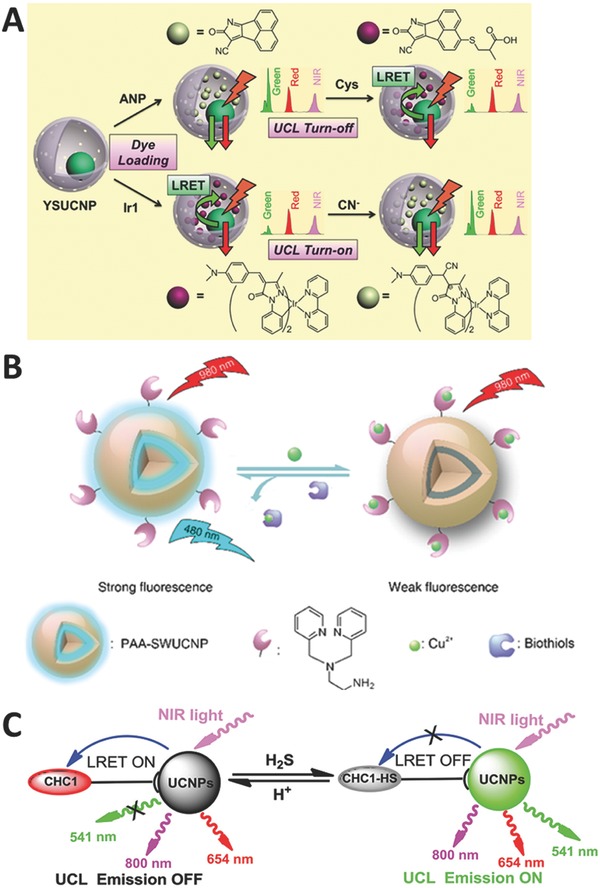
Schematic illustration for detection of A) Cys (Reproduced with permission.^116^ Copyright 2014, American Chemical Society), B) GSH (Reproduced with permission.^121^ Copyright 2016, American Chemical Society), and C) H_2_S (Reproduced with permission.^123^ Copyright 2014, Wiley‐VCH Verlag GmbH & Co. KGaA).

Li and co‐workers developed a yolk–shell‐structured UCNP probe for the detection of Cys and Hcy with organic dyes as energy acceptors.^116^ The hollow cavity of the yolk–shell structure is utilized to accommodate the energy acceptors as guest molecules (**Figure** **7**A). This strategy does not require any specific chemical modifications to the probe. Moreover, this protocol could be improved as a common approach to construct other UCNP hybrid probes with different dyes. Recently, Fan and co‐workers employed AgNPs as energy acceptors,^117^ and Li and co‐workers presented rhodamine–oxaldehyde dyes^118^ and fluorescein‐based dyes^119^ as energy acceptors to detect Cys, respectively.

Min and co‐workers integrated UCNPs with magnetic particles to construct nanohybrid probes for the detection of GSH.^120^ They encapsulated UCNPs and Fe_3_O_4_ into a silica matrix, and decorated with AuNPs as energy acceptors through disulfide bond. GSH will break the disulfide bond and turn on the emission. Liu and co‐workers presented a new approach to construct upconversion probes through heavy metal‐ion‐induced quenching.^121^ The commonly used LRET process requires a spectral overlap, leading to limited flexibility in probe design and hindering the construction of upconversion probes. To improve this strategy, in another study, Cu^2+^ was employed as energy quenchers for GSH detection with a satisfied performance both in in vitro test and bioimaging (Figure 7B). The as‐fabricated probe shows that heavy metal ions could quench more than 95% of the UCL without the occurrence of spectral overlap. In addition, it is the first time the readout signal of upconversion is manipulated through heavy metal ions. Chen and co‐workers reported another UCNP probe for the detection of Cys and GSH through a reduction reaction,^122^ where they attach Fe^3+^ onto the surface of UCNPs, and Fe^3+^ can be further reduced into Fe^2+^ by Cys and GSH, leading to the turn‐on of the emission.

Although H_2_S is a toxic gas with unique foul odor of rotten eggs, small amount of H_2_S in human bodies is very important because it can act as a biological signaling molecule in biosystem. Zhang and co‐workers reported a dye‐modified UCNP probe to detect H_2_S with a fast response time and a large ratiometric UCL enhancement.^123^ In their research, they employ coumarin–hemicyanine (CHC1) as an energy acceptor, and the addition of H_2_S can trigger the nucleophilic addition to merocyanine and turn on the emission (Figure 7C). This probe could be applied in monitoring pseudoenzymatic H_2_S production in living cells, and identifying the risk of endotoxic shock as well. Zhao and co‐workers fabricated a novel core–shell UCNP probe for the detection and bioimaging of intracellular H_2_S,^124^ where the mesoporous mSiO_2_ can adsorb merocyanine as an energy acceptor. Chang and co‐workers developed a powerful platform for H_2_S detection with high selectivity and rapid response time in vitro and in vivo.^125^ They prepared a series of dyes with different absorption bands. After combined with UCNPs, a library of H_2_S probes with emission signals ranging from visible to near‐infrared region is obtained.

## 4 Conclusion

UCNPs have many special optical properties that could make UCNP‐based probes suitable in biodetection. In this review, the main upconversion mechanisms and LRET process are discussed. Changing spectral overlap and distance between UCNPs and energy acceptors are two key approaches to manipulate LRET efficiency and achieve the efficient detection goal in biosamples. Moreover, various studies have been summarized for the detection of inorganic ions, pH, gas molecules, reactive oxygen species, and thiols **Table** **1**. In these studies, most of these probes employ organic dyes, quantum dots, graphene oxide, and noble metal nanoparticles as energy acceptors.

**Table 1:** Detection results for different analytes. For the detection strategy column, both directions (turn on or turn off) are classified in same strategy

Analyte	Energy acceptor	Limit detection/detection range	Strategy	Excitation wavelength [nm]	Ref.
Ag^+^	Organic dye	33 × 10^−9^ m	Scheme 1A	980	53
	Quantum dots	60 × 10^−12^ m	Scheme 1B	980	54
Ca^2+^	Organic dye	15 × 10^−12^ m	Scheme 1B	980	55
Fe^3+^	Organic dye	89.6 × 10^−9^ m	Scheme 1A	980	56
Zn^2+^	Organic dye	–	Scheme 1A	980	57
	Organic dye	0.18 × 10^−6^ m	Scheme 1A	980	58
Cu^2+^	Organic dye	2.16 × 10^−6^ m	Scheme 1A	980	59
	Organic dye	0.82 × 10^−6^ m	Scheme 1A	980	60
	Organic dye	–	Scheme 1A	980	61
	Organic dye	57.8 × 10^−9^ m	Scheme 1A, 1B	980	62
Cr^3+^	Organic dye	4.1 × 10^−6^ m	Scheme 1A	980	63
Hg^2+^	Organic dye	8.2 ppb	Scheme 1A	980	64
	Organic dye	0.21 × 10^−6^ m	Scheme 1A	980	65
	Organic dye	0.16 × 10^−6^ m	Scheme 1A	980	66
	Quantum dots	15 × 10^−9^ m	–	980	67
	Quantum dots	70.5 × 10^−9^ m	–	980	68
	Heavy metal ions	5 × 10^−9^ m	Scheme 1C	980	69
	Au nanoparticles	150 × 10^−12^ m	Scheme 1D	980	76
	Organic dye	–	Scheme 1A	980	92
Cd^2+^	Au nanoparticles	0.2 × 10^−6^ m	Scheme 1C	980	73
Pb^2+^	Quantum dots	80 × 10^−9^ m	–	980	74
	Au nanoparticles	20 × 10^−9^ m	Scheme 1B	980	75
	Au nanoparticles	50 × 10^−12^ m	Scheme 1D	980	76
	Carbon nanohorns	9.7 × 10^−9^ m	Scheme 1D	980	77
	Graphene oxide	10.8 × 10^−9^ m	Scheme 1D	980	77
CN^−^	Organic dye	0.84 × 10^−6^ m	Scheme 1A	980	78
	Organic dye	6.7 × 10^−6^ m	Scheme 1A	980	116
F^−^	Organic dye	5 × 10^−6^ m	Scheme 1A	980	79
NO_2_^−^	Organic dye	16.1 × 10^−6^ m	Scheme 1A	980	81
pH	Organic dye	6.0–8.0	Scheme 1A	980	83
	Organic dye	–	Scheme 1A	980	84
	Organic dye	3.0–6.7	Scheme 1A	980	85
	Organic dye	5.0–7.0	Scheme 1A	980	86
	Organic dye	–	Scheme 1A	980	87
	Organic dye	–	Scheme 1A	980	88
	Graphene oxide	5.0–8.0	–	980	89
	Organic dye	4.0–8.0	Scheme 1A	980	90
	Organic dye	3.0–7.0	Scheme 1A	980	91
	Organic dye	–	–	980	92
O_2_	Organic dye	–	Scheme 1A	980	94
	Organic dye	–	Scheme 1A	980	95
	Organic dye	–	Scheme 1A	980	96
	Organic dye	–	Scheme 1A	980	97
NO	Organic dye	73 × 10^−9^ m	Scheme 1A	980	98
H_2_O_2_	Organic dye	0.08 × 10^−6^ m	Scheme 1A	980	102
	MnO_2_ nanosheet	0.9 × 10^−6^ m	Scheme 1B	980	103
	Ag nanoparticles	1.08 × 10^−6^ m	Scheme 1B	980	104
	Organic dye	45 × 10^−9^ m	Scheme 1A	980	105
	Organic dye	0.8 × 10^−6^ m	Scheme 1A	980	106
•OH	Organic dye	1.2 × 10^−15^ m	Scheme 1A	980	109
	Organic dye	0.21 × 10^−6^ m	Scheme 1B	980	110
	Organic dye	4 × 10^−12^ m	Scheme 1A	980, 808	111
HClO	Organic dye	0.32 × 10^−6^ m	Scheme 1A	980	112
	Organic dye	3 × 10^−9^ m	Scheme 1B	980	113
	Organic dye	27 ppb	Scheme 1A	808	114
Cys	Organic dye	28.5 × 10^−6^ m	Scheme 1A	980	116
	Ag nanodisks	–	Scheme 1B	980	117
	Organic dye	1.1 × 10^−6^ m	Scheme 1A	980	118
	Organic dye	20 × 10^−6^ m	Scheme 1A	980	119
	Heavy metal ions	0.5 × 10^−6^ m	Scheme 1B	980	122
GSH	Au nanoparticles	5 × 10^−6^ m	Scheme 1B	980	120
	Heavy metal ions	7.2 × 10^−9^ m	Scheme 1B	980	121
	Heavy metal ions	0.2 × 10^−6^ m	Scheme 1B	980	122
H_2_S	Organic dye	0.13 × 10^−6^ m	Scheme 1A	980	123
	Organic dye	0.58 × 10^−6^ m	Scheme 1A	980	124
	Organic dye	–	Scheme 1A	980	125

Although significant progress in this research field has been witnessed, there still remain several issues required to be addressed. First, the relatively low upconversion efficiency is a big challenge, which will limit their final applications in human body. Developing efficient core–shell structures might be a promising way to reduce the surface quenching effect. One should be careful that the outside shell may increase the distance between UCNPs and energy acceptors, leading to a lower LRET efficiency. Thus, more efforts are required to find an optimized solution. The second issue is biocompatibility. Usually, the as‐prepared UCNPs are covered with hydrophobic layer. Thus, an effective strategy to modify the surface of UCNPs is required to make them water soluble. The third challenge is how to develop ultrasmall (less than 10 nm) UCNP‐based probes. Ultrasmall UCNPs could easily penetrate biosamples without any degradation. Clearly, such probes are more suitable for biodetection and bioimaging. However, there is a considerable loss of emission efficiency due to the surface quenching effect. This issue should be addressed in the future. In brief, UCNP‐based probes should have their own potentials in bioimaging and biodetection; however, there remains a large room for the improvement before they could find real practical applications.

## Conflict of Interest

The authors declare no conflict of interest.
